# Multi-task learning for Chinese clinical named entity recognition with external knowledge

**DOI:** 10.1186/s12911-021-01717-1

**Published:** 2021-12-31

**Authors:** Ming Cheng, Shufeng Xiong, Fei Li, Pan Liang, Jianbo Gao

**Affiliations:** 1 Department of Medical Information, The First Affiliated Hospital of Zhengzhou University, Zhengzhou, China; 2 Colleges of Information and Management Science, Henan Agricultural University, Zhengzhou, China; 3 School of Cyber Science and Engineering, Wuhan University, Wuhan, China; 4 Department of Radiology, The First Affiliated Hospital of Zhengzhou University, Zhengzhou, China

**Keywords:** Chinese clinical named entity recognition, Multi-task learning, Deep neural network, Dictionary features

## Abstract

**Background:**

Named entity recognition (NER) on Chinese electronic medical/healthcare records has attracted significantly attentions as it can be applied to building applications to understand these records. Most previous methods have been purely data-driven, requiring high-quality and large-scale labeled medical data. However, labeled data is expensive to obtain, and these data-driven methods are difficult to handle rare and unseen entities.

**Methods:**

To tackle these problems, this study presents a novel multi-task deep neural network model for Chinese NER in the medical domain. We incorporate dictionary features into neural networks, and a general secondary named entity segmentation is used as auxiliary task to improve the performance of the primary task of named entity recognition.

**Results:**

In order to evaluate the proposed method, we compare it with other currently popular methods, on three benchmark datasets. Two of the datasets are publicly available, and the other one is constructed by us. Experimental results show that the proposed model achieves 91.07% average f-measure on the two public datasets and 87.05% f-measure on private dataset.

**Conclusions:**

The comparison results of different models demonstrated the effectiveness of our model. The proposed model outperformed traditional statistical models.

## Introduction

With rapid development of Electronic Medical Records (EMRs) systems, there has been an increasing interest in applying text mining and information extraction to the EMRs. Those techniques can generate tremendous benefits for both medical research and applications. Among the medical texts mining tasks, NER is a fundamental task which locates the mentions of named entities and classifies them (e.g. symptoms, tests, drugs, operations and diseases, etc.) in unstructured medical/healthcare records [1–4]. However, learning clinical entities in medical domain is a challenging task: (1) various non-standard expressions, multiple variants of the same entity, often appeared in clinical records, and (2) the sentence structure of clinical records are often incomplete, with less context and more grammatical errors [5].

Recently, deep learning approaches achieved state-of-the-art performance in NER tasks [6–8]. However, the deep models usually require a large amount of labeled data for training, while manual annotation is time-consuming. In order to alleviate the dependence of large annotation data, some researchers proposed to integrate prior knowledge into the models [9]. One possible solution is incorporating dictionary feature into the models. Wang et al. [10] showed the effectiveness of using dictionary features in the BiLSTM model, which is conducive to better process the rare clinical entities.

**Fig. 1:**
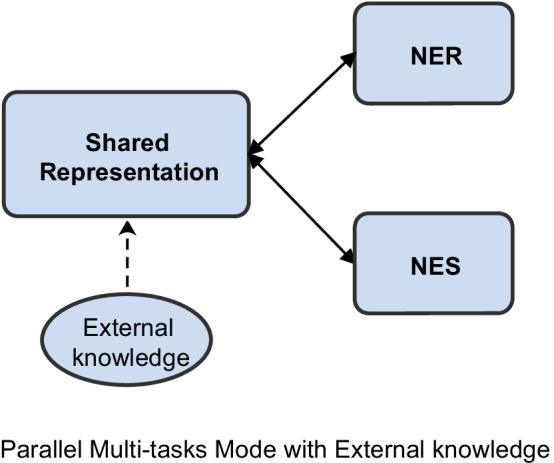
Parallel multi-task model by incorporating external knowledge and shared representation among tasks

Inspired by the observations, we propose a Dictionary-based Multi-Task neural network model called DicMT, as shown in Fig. 1. In addition to the primary entities recognition task, we also use an auxiliary but related secondary tasks Named Entity Segmentation (NES). The NES is a binary classification task, its goal is to predict whether or not a token is part of an entity. We use these two tasks to jointly train the network. Moreover, to cover more clinical entities, we design five n-gram feature templates to construct dictionary features. Finally, we conducted generous experimental evaluation for the proposed approach on three medical datasets.

The main contributions of this article are as follows:

1. We present a multi-task learning framework which jointly trains a model to perform entity segmentation with cross-entropy loss and entity recognition task with CRF.
2. We make use of the dictionary information by incorporating the dictionary features into deep neural network, for enhancing the recognition of rare entities.
3. A Chinese pre-trained BERT model based on Chinese EMRs is constructed, which can be used to other Chinese medical text mining tasks.

Although there are some similar works about external knowledge, our work is different from them as follows.

- To the best of our knowledge, it is the first time that the dictionary features have been integrated into a multi-task learning framework for the clinical NER task. And we devise five n-gram templates to extract dictionary features.
- Our work aims to study the integration mechanism of dictionary features into multi-task deep learning models, rather than simply enhancing the model performance.

The rest of this article is organized as follows. “Related work” section briefly reviews the related work on clinical NER. In “Materials and methods” section, we describe our proposed approach. In “Results” section, we present the model results and evaluate the performance of the model in three datasets. The experimental results and the limitations of this work are discussed in “Discussion” section. Finally, “Conclusion” section contains conclusions and suggestions for future research directions.

## Related work

Clinical NER has become an important research field in medical information extraction and healthcare data mining [11, 12]. The development of clinical NER has basically undergone a transformation from rules to deep learning technology, mainly including the following methods.

Traditionally, rule-based approaches use heuristic rules to identify named entities. Based on the characters/words themselves and their contexts the heuristic rules were used to learn recognition patterns [13, 14], such that in clinical texts, the phrases ending with “  (operation)” indicates operations and the character “  (cancer)” could be regarded as the end of disease tokens. But the handcrafted rules are commonly limited, it is hard to list all the entity extraction rules, and it is also difficult to translate from one field to another. Dictionary-based methods commonly rely on the vocabularies information contained in it to match the entities in the clinical records [15–17]. However, for entities not listed in the dictionary, it usually fails to process, resulting in low recalls.

Statistical machine learning approaches such as maximum entropy models [18], Conditional Random Fields (CRF) [19], and hidden markov models [20], they treat the NER as a sequence labeling task, and use a amount of annotated data to learn tagging models [21]. The statistical machine learning approaches depend on the pre-defined features template, which makes modeling process more costly. The feature templates are usually composed of several handcrafted features, while the best feature set need conduct a lot of trial-and-error experiments [22].

Recently, deep learning techniques have been demonstrated to be the most advanced performance in many areas. Some researchers had proposed Long Short-Term Memory (LSTM-CRF) model for sequence tagging, which is a combination of feature templates and neural network. The Bi-directional Long Short-Term Memory (BiLSTM) is further developed into a LSTM-CRF as presented in [23, 24], where the dependency between nodes in the output layer is explicitly captured by a CRF-like chain. Ma et al. [25] presented a neutral network architecture which combines character- and word-level representations and feed them into BiLSTM-CRF model for sequence labelling tasks. Khan et al. [26] proposed a disease NER model which intergates the contextual embeddings with relevant domain-specific features, character and word embeddings into a BiLSTM-CRF framework. Sahu et al. [27] proposed a disease NER model that cascades a Convolution Neural Network (CNN) model and a Recurrent Neural Network (RNN) to get character embeddings. Dong et al. [28] proposed a LSTM-CRF architecture which has a radical LSTM layer to learn the radical features of characters from the annotated corpus.

Neural multi-task learning is a learning paradigm in machine learning. Its purpose is to employ the useful information contained in multiple related tasks to improve the performance of all tasks [29]. It has been used successfully across many tasks of NLP [30]. Fei et al. [31] used multi-task learning framework to identify the nested medical entities in biomedical texts. Wang et al. [32] proposed a multi-task learning framework for biomedical NER, which emploied training data of different entity types to improve the performance of each entity. However, few studies have explored how to combine multi-task learning framework with external knowledge. It is important to investigate whether this combination is more effective than the traditional methods. In the present study, we systematically evaluated the performance of our method in three Chinese clinical datasets.

## Materials and methods

**Fig. 2:**
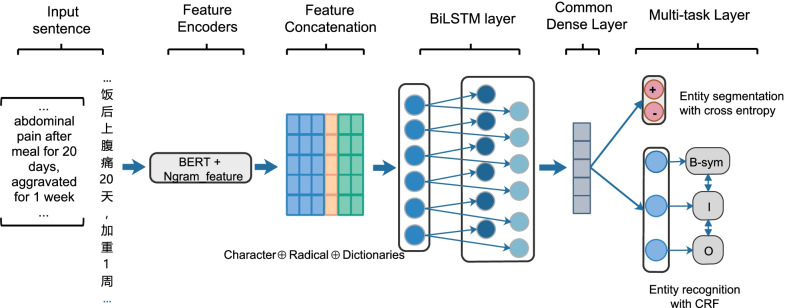
The framework of our system. First, the system embeds a sentence into a high dimensional space to extract features. Then, it concatenates the resulting vectors of each encoder and performs multi-task. The right pink nodes layer represents segmentation while the right blue nodes layer represents categorization

The Chinese clinical NER task is usually known as a sequence labelling task, while NES task is considered as binary classification task of whether a token is entity or not. In order to make the most of the mutual benefits between NER and NES, we propose a dictionary-based multi-task neural network, the whole framework of our system can be found in Fig. 2.

Moreover, we label the sequence on the character-level. Formally, given a Chinese clinical sentence $X=x_0, \dots , x_n$, we employ the BIO (Begin, Inside, Outside) tag scheme to tab each character $x_i$ in the sentence *X*, i.e. generating a tag sequence $Y=y_1,\dots ,y_n$. An example of the sequence labeling for “  ” (Nausea after a meal for more than half a year, abdominal pain after a meal for 5 days, worse for 1 week) can be found in Table 1. The B-tag and I-tag indicate the beginning and inside of an entity, respectively. And, the O-tag indicates that the character is outside an entity. For entity segmentation task, “1” indicates that a token is part of the entity, or “0” otherwise.

**Table 1:**
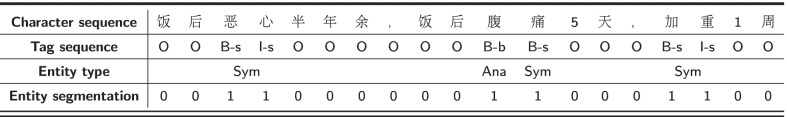
An illustrative example of the tag sequence

“Sym” is an abbreviation for “Symptom”, and “Ana” is an abbreviation for “Anatomy”

### Feature representation

The features representation are divided into two categories: Chinese characters and external dictionary.

#### Chinese character representation

Bidirectional Encoder Representations from Transformer (BERT, https://github.com/google-research/bert/) has shown great performance improvements in various NLP tasks, which uses a mount of unannotated data and generates rich contextual representations. In this section, our purpose is to build a Chinese pre-trained BERT model based on a large collection of unlabelled Chinese clinical records from the first affiliated hospital of Zhengzhou University, and made the pretrained model available on our experiments. In addition, we advance radicals-level features for Chinese characters to capture its pictographic root features.

We obtained 7.8G original electronic medical records from the first affiliated hospital of Zhengzhou University. All sensitive information has been deleted, including name, ID, telephone, address, hospitalization number, etc. Only the main complaint, diagnosis and treatment process are adopted. After data preprocessing, we obtain 1.2G clinical records. The corpora consisted of different medical domain, including gastrointestinal surgery, cardiovascular, gynaecology, orthopaedics etc. For pre-training BERT, similarly with paper [33], based on the existing BERT checkpoint we run additional pre-training steps on the specific domains to fine-tune BERT model.

The Chinese characters usually consist of smaller substructure, called radicals. These radicals have the potential characteristics of Chinese characters and bring additional semantic information. The Chinese characters’ written form often share a common sub-structures and some of these sub-structures are same semantic information. For example, the characters “  ” (liver), “  ” (gland), “  ” (abdomen) all have the meaning related to “  ” (meat) because of their shared sub-structure “  ”, a simplified form of traditional radical “  ” (meat). Inspired by these observations, we add radical feature to character representation.

#### External dictionaries representation

In the previous work, the dictionary information have been considered to be useful in clinical NER task [10]. Here, we adopt similar dictionary feature encoding scheme in Wang and Zhou’s work [10], n-gram scheme to represent dictionary information. Given a sentence *X* and some external dictionaries *D*, based on the context of $x_i$, we adopt the pre-defined n-gram features templates to construct text fragments. Table 2 lists all n-gram templates.

**Fig. 3:**
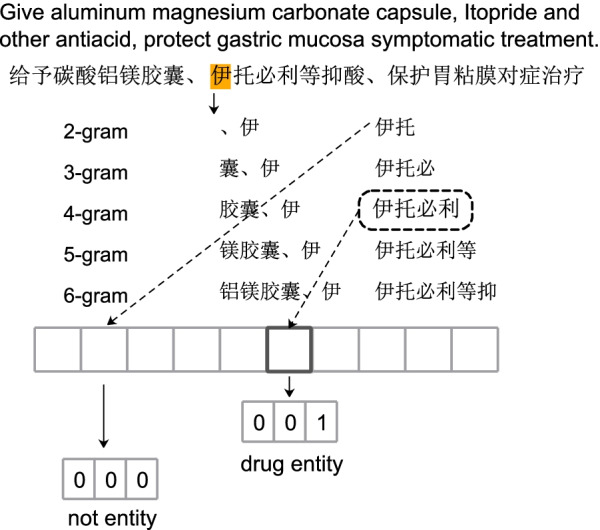
An illustration for n-gram feature construction. An segment sample of the drug entity with solid rectangle. The character $x_i$ is represented by the yellow shadow

The n-gram feature template generated ten text fragments. For these text fragments, we design five binary vectors to represent different clinical entity types in *D*. In CCKS2017 dataset, the disease entity is represented as (0, 0, 1), anatomy (0, 1, 0), symptom (0, 1, 1), exam (1, 0, 0), treatment (1, 0, 1). In CCKS2018 and FCCd dataset, the drug entity is represented as (0, 0, 1), anatomy (0, 1, 0), independent symptom (0, 1, 1), describe symptom (1, 0, 0), operation (1, 0, 1). And (0, 0, 0) indicates this text segment is not an clinical entity. Here we use $t_{i,j}$ to indicate the output in *j*th n-gram template for $x_i$. Finally, we generate a 30-dimensions dictionary feature vector for $x_i$, which contains types of entities and boundary information between characters. Figure 3 shows an illustrative example of n-gram feature generation.

**Table 2:** N-gram feature templates of the *i*th character

Types	Template
2-gram	$x_{i-1}x_i, x_ix_{i+1}$
3-gram	$x_{i-2}x_{i-1}x_i, x_ix_{i+1}x_{i+2}$
4-gram	$x_{i-3}x_{i-2}x_{i-1}x_i, x_ix_{i+1}x_{i+2}x_{i+3}$
5-gram	$x_{i-4}x_{i-3}x_{i-2}x_{i-1}x_i, x_ix_{i+1}x_{i+2}x_{i+3}x_{i+4}$
6-gram	$x_{i-5}x_{i-4}x_{i-3}x_{i-2}x_{i-1}x_i, x_ix_{i+1}x_{i+2}x_{i+3}x_{i+4}x_{i+5}$

### Multi-task network

Clinical named entities segmentation and recognition are two related tasks and their outputs potentially have mutual benefits for each other as well. Specifically, the output of NES could reduce the searching space of NER and vice versa. Therefore, We present a multi-task learning framework to train clinical entities segmentation and recognition model simultaneously while sharing parameters through these models. In addition, we exploit BiLSTM to power the sequential modeling of the text, as shown in Fig. 2.

The extracted features of each character, including pre-trained character embedding from fine-tune BERT, radical-level and dictionary features, are fed into a bidirectional long short-term memory networks. The output of network at each time step is jointly decoded the best chain of labels by a linear layer and a CRF layer. For each position *t*, LSTM computes $h_t$ with input $x_t$ and previous state $h_{i-1}$, we use the following implementation:

1 $$\begin{aligned} \begin{aligned} i_t&= \lambda (W_{xi}x_t + W_{hi}h_{t-1} + b_i) \\ f_t&= \lambda (W_{xf}x_t + W_{hf}h_{t-1} + b_f) \\ c_t&= f_t \odot c_{t-1} + i_t \odot tanh(W_{xc}x_t+W_{hc}h_{t-1}+b_c) \\ o_t&= \lambda (W_{xo}x_t + W_{ho}h_{t-1} + b_o) \\ h_t&= o_t\odot tanh(c_t) \end{aligned} \end{aligned}$$

where $x_t$ is the input vector at time *t*, the $\lambda$ is the element-wise sigmoid function. $h_t$ is the hidden state vector, *W* are weight matrices, *b* are biases, and $\odot$ denotes the element-wise multiplication. Finally, the both forward and backward hidden states are concatenated for a final representation $[\overrightarrow{h_i}; \overleftarrow{h_i}]$.

Formally, given a Chinese clinical sentence $X = x_0x_1\dots x_n$, where $x_t (1\le t \le n)$ is the *t*th Chinese character, we follow $x_t$ by $[p_{t}\oplus r_t \oplus d_{t}]$, where $p_t, r_t$ and ${d_{t}}$ are pre-trained character embedding, radical-level features and its dictionary features respectively, and $\oplus$ is the concatenation operation, such as Fig. 2.

Typically, the additional auxiliary task is used as a regularizer to generalize the model. For the binary classification task of entity segmentation, the sigmoid activation function and cross-entropy loss are be used, whereas for the primary entity recognition task, we adopt CRFs layer to predict the possible labels.

Furthermore, we use the weights learned from the common layer to capture the generalization features of two tasks. Then, the learned weights were used as input for the CRFs layer (see Fig. 2). Finally, the total losses of the two tasks were fed backward during the training process.

### Training objective

#### The entity segmentation with cross-entropy loss

For the binary classification task of entity segmentation, the cross-entropy loss was used. The entities are labelled “1” and non entities are labelled “0”.

Suppose that *p* is the one-hot true probability distribution for all classes $C=\{c\}$, and *q* is the predicted probability distribution. The cross-entropy loss of a instance can be expressed as:

2 $$\begin{aligned} H(p,q) = -\sum _{c\in C}p(c)log(q(c)) \end{aligned}$$

So the loss function of this task would be:

3 $$\begin{aligned} loss_1 = -\sum (p(1)log(q(1))+p(0)log(q(0))) \end{aligned}$$

#### The entity recognition with CRFs

Since CRFs considers the correlations between labels in neighborhoods and jointly decodes the best chain of labels for a given input, we model label sequence jointly using a CRFs to predict the possible tags.

Formally, the inputs of CRFs is the hidden output *z*. The probabilistic model for sequences CRFs defines a family of conditional probability *p*(*y*|*z*; *W*, *b*) over all possible label sequences *y* given *z* by the following formulation:

4 $$\begin{aligned} p(y|z;W,b) = \frac{\prod _{i=1}^{n}\psi _i(y_{i-1},y_i,z)}{\sum _{y^*\dot{\in }Y(z)}\prod _{i=1}^n\psi _i(y^*_{i-1},y_i^*,z) } \end{aligned}$$

where $\psi _i(y^*_{i-1},y_i^*,z)=exp(W^T_{y^*,y}z_i+b_{y^*,y})$ are potential functions, $W^T_{y^*,y}$ and $b_{y^*,y}$ are the weight vector and bias corresponding to label pair ($y^*, y$), respectively.

CRFs layer is trained under the maximum conditional likelihood estimation. For a training set $(z_i,y_i)$, the logarithm of the likelihood is given by:

5 $$\begin{aligned} loss_2(W,b) = \sum _ilogp(y|z;W,b) \end{aligned}$$

We directly combine the losses of all individual tasks as the multi-task setting. Moreover, we introduce the regulating factors $\alpha$ and $\beta$ to balance the loss of the two tasks. Finally, we feed the total loss from both tasks backward during training. The total loss of multi-task framework can be defined as:

6 $$\begin{aligned} L = \alpha \cdot loss_1 + \beta \cdot loss_2 \end{aligned}$$

where $\alpha$ and $\beta$ are weights for the losses of two tasks. Our training objective is to jointly optimize the common network parameters.

### Prediction

We only use the output of CRFs to make predictions. Decoding process based on viterbi algorithm is used to search for a label sequence $y^*$ with the highest conditional probability:

7 $$\begin{aligned} y^* = argmin_{y\in Y} p(y|z;W,b) \end{aligned}$$

Finally, CRFs computes a structured output sequence $Y = \{ y_1,\ldots ,y_n \}$.

## Results

The dictionary was constructed in the experiments according to the lists of operation information, drug information and charging items of the first affiliated hospital of Zhengzhou University.

We evaluate our method on three datasets: CCKS2017, CCKS2018 and FCCd. The CCKS2017 (http://www.ccks2017.com/) designed five clinical entities types (anatomy, symptom, disease, exam, treatment) based on 1596 Chinese admission records. 1198 of the records of them are used as a training set, 398 records are test set. The total number of clinical entities is 39359. The CCKS2018 (http://www.ccks2018.cn/) designed five clinical entities types (anatomy, independent symptom, symptom description, operation, drug) based on 600 Chinese admission records. 500 of the records of them are used as a training set, 100 records are test set. The total number of clinical entities is 11980. In addition, we construct a real medical dataset from the first affiliated hospital of Zhengzhou University (FCCd, 736 discharge records). The 609 records are used as training set, and 127 are test set. We identified 5 categories of clinical entities: “Anatomy”, “Operation”, “Drug”, “Independent symptoms”, “Describe symptoms”. We only annotated continuous entities, which are independently annotated by two medical students. If there is a difference in the labeling process, an experienced clinician is responsible for dealing with the inconsistencies between the two annotations. The total number of entities is 19133. Table 3 lists the statistics of the three datasets.

We use widely-used evaluation strategies, namely recall, precision and f-measures to evaluate our method in the experiments [33–35]. F-measure is the harmonic mean of precision and recall.

8 $$\begin{aligned} F-measure=\frac{2\times recall \times precision}{recall+precision} \end{aligned}$$

**Table 3:** Statistics of the entity recognition in Chinese clinical texts

**CCKS2017**	**Symptom**	**Disease**	**Exam**	**Treatment**	**Anatomy**	**All**
Total (1596)	10142	1275	12689	1513	13740	39359
**CCKS2018**	**Anatomy**	**Operation**	**Drug**	**IndeSym**	**DesSym**	**All**
Total (600)	5574	1085	849	2764	1708	11980
**FCCd**	**Anatomy**	**Operation**	**Drug**	**IndeSym**	**DesSym**	**All**
Total (736)	9686	1164	1105	4117	3061	19133
**Total**	Records (2932)			Clinical Entities (70472)		

### Experimental setup

The parameter configurations are shown in Table 4. In our experiments, we used dropout training with a probability of 0.5 to avoid overfitting. The Adam algorithm was used to optimize the training, and the initial learning rate is 0.0005. We exploit the Chinese full stop “  ” to separate the medical records for restricting the sentence length. After cutting records, the length of sequences is padded to 250 in three datasets. The regulating factors $\alpha$ and $\beta$ can be fine-tuned through experiments. In our experiments, we set $\alpha {:}\beta =2{:}3$ which may yield the best result. All experiments are carried out by using two GTX2080Ti GPUs with 11GB memory.

**Table 4:** Parameters of our model in the experiments

**Parameters**	**Value**
Dim of character embedding	100
Dim of radical embedding	50
Number of BiLSTM hidden units	128
Dropout	0.5
Batch size	32
Epochs	300

### Compared with state-of-the-art models

To show the effectiveness of the proposed model, we used the following methods as baselines:

- **Wang** et al. [10]: an method for integrating token-level dictionary features into the deep neural model for entity recognition.
- **Hu** et al. [36]: a hybrid method for entity recognition.
- **Zhang** et al. [37]: combining multi-task framework, self-attention and multi-step training methods to develop more features for entity recognition task.
- **Qiu** et al. [38]: a residual dilated convolutional neural network with conditional random field for clinical named entitiy recognition, RD-CNN-CRFs.
- **Li** et al. [33]: the variant neural structures based on BERT methods for clinical named entity recognition.
- **Tang** et al. [23]: another extended version of LSTM-CRFs, which added CNN layer and attention layer to develop performance of entity recognition, called attention-based CNN-LSTM-CRFs.
- **Luo** et al. [39]: a neural network ensemble approach.
- **Yang** et al. [40]: a conditional random fields (CRFs) model based on different features.

Table 5 shows experimental results of different models on CCKS2017, CCKS2018 and FCCd. We can see that the proposed multi-task framework could achieve the best performance, outperforming state-of-the-art systems. The multi-task learning mechanism can obtain more dependency information. Moreover, the results of experiments indicate that incorporating the dictionary and radical-level features on multi-task neural network architecture is effective. The main reasons are that (1) the pre-trained BERT on a large Chinese EMRs could obtain better character representations comparing to traditional methods; (2) The additional dictionary contains rare entities, our method could handle them better than former methods; (3) The different Chinese clinical entities usually share the same radicals, such as “  (liver)”, “  (spleen)” and “  (abdominal cavity)” they all shared same radical “  ”, etc. These additional radical-level features can benefit recognition.

**Table 5:** Comparative results with F-measure between different models on three datasets

Method	CCKS2017	CCKS2018	FCCd
Wang et al. [10]	91.24	89.72	86.07
Hu et al. [36]	91.03	–	–
Zhang et al. [37]	90.52	–	–
Qiu et al. [38]	91.32	–	–
Li et al. [33]	91.60	89.56	86.87
Tang et al. [23]	90.61	88.63	86.24
Luo et al. [39]	91.36	88.63	85.52
Yang et al. [40]	90.16	89.13	84.73
Our	91.84	90.29	87.05

### Ablation study

Our model contains several parts, and it is important to understand the influence of different parts on performance. The ablation research aims to explore the influence of character embeddings, dictionary information and multi-tasking learning on the model. We conduct experiments on two datasets, CCKS2017 and FCCd.

#### Impact of different character embeddings

We compare the effects of different character embeddings on the performance of the model. Our proposed multi-task learning framework was used as base network. The firstly, the random initialized 100-dimensional embeddings is used as character embedding, which are uniformly sampled from range $[-\sqrt{\frac{3}{dim}},+\sqrt{\frac{3}{dim}}]$, where *dim* is the dimension of embeddings. The secondly, we use the BERT model (Baseline1) trained on the Chinese corpus of general field as baseline model. In addition, the fine-tuned BERT model (Baseline2) which is fine-tuned on Chinese clinical corpora is as a baseline model as well. The last, we add radical-level features to capture the pictographic root features of Chinese character. The dimension of radical embeddings is 50. The experiment results can be seen in Table 6.

In order to explore the impact of different character embeddings in our method, we remove one or two of them from our network, and show the results in Table 6, where precision, recalls and f-measures are listed, “BERT” denotes BERT model trained on general domain. “FT-BERT” denotes fine-tuned BERT on special Chinese clinical corpus. “FT-BERT-radical” denotes Chinese character embedding based on radical-level and FT-BERT. The best result is in bold (the following sections also use the same way to denote the best result).

The BERT model f-measures 90.62% is higher than random embedding on two datasets. The performance of FT-BERT is significantly better than that of the BERT. After adding radical features to FT-BERT model, the f-measures is slightly higher than FT-BERT model on CCKS2017. On both two datasets, the best architecture is based on the FT-BERT + radical features, which improves the f-measures compared with other methods. The experimental results shows the radical features and FT-BERT trained on Chinese clinical corpora can improve the performance. We apply radical + FT-BERT to multi-task learning framework can achieve 91.51% of F-measure, which outperforms the baseline models.

**Table 6:** Impact of the different character embeddings in our method

Dataset	Method	Precision	Recall	F-measure
CCKS2017	Random	89.56	89.29	89.42
	BERT(Baseline1)	90.73	90.51	90.62
	FT-BERT(Baseline2)	91.27	91.21	91.24
	FT-BERT-Radical	91.69	91.34	**91.51**
FCCd	Random	84.23	83.32	83.77
	BERT(Baseline1)	86.11	85.52	85.81
	FT-BERT(Baseline2)	86.21	85.83	86.02
	FT-BERT-Radical	86.95	86.56	**86.75**

The best result is in bold

#### Impact of dictionary information

We investigate the contribution of dictionary information to model performance by adding dictionary features. After adding dictionary features, the performance is significantly improved. The dictionary features can effectively identify the rare entities, which proves that dictionary features are meaningful. This is consistent with the results in Table 7.

**Table 7:** Impact of the dictionary features on our method

Dataset	Method	Precision	Recall	F-measure
CCKS2017	FT-BERT-Radical	91.69	91.34	91.51
	FT-BERT-Radical+Dictionary	91.91	91.78	**91.84**
FCCd	FT-BERT-Radical	86.95	86.56	86.75
	FT-BERT-Radical+Dictionary	87.32	86.79	**87.05**

The best result is in bold

In order to investigate effects of the dictionary information in our method, we conduct experiments to analyze impact of dictionary size on performance of model. We construct four new sub-dictionaries by randomly select 70%, 80%, 90%, 100% of the entities from the original dictionary. The experimental results were shown in Table 8.

**Table 8:** Impact of the different dictionary sizes on method performance

Dataset	Dictionary size	Precision	Recall	F-measure
CCKS2017	70%	91.79	91.61	91.70
	80%	91.83	91.69	91.76
	90%	91.87	91.73	91.80
	100%	91.91	91.78	**91.84**
FCCd	70%	87.19	86.65	86.92
	80%	87.23	86.71	86.97
	90%	87.28	86.75	87.01
	100%	87.32	86.79	**87.05**

The best result is in bold

From Table 8, as the dictionary size increases, the performance of our method gradually improves. The experimental results indicate that the more clinical entities the dictionary contains, the better the performance of the model is.

#### Impact of w/o multi-tasking

In order to investigate the impact of multi-tasking framework to our model, we compare the performance of the networks with and without multi-tasking based on the same feature representations and dictionary information, as shown in Table 9. Reducing the multi-tasking framework, our model degenerates to the basic BiLSTM-CRF networks.

**Table 9:** Performances of the networks with and without multi-task learning on the two datasets

Dataset	Method	Precision	Recall	F-measure
CCKS2017	Single-task for NES	92.06	91.55	91.80
	Single-task for NER	91.72	91.59	91.65
	Multi-task for NER	91.91	91.78	**91.84**
FCCd	Single-task for NES	87.24	86.81	87.02
	Single-task for NER	87.03	86.60	86.81
	Multi-task for NER	87.32	86.79	**87.05**

The best result is in bold

Table 9 shows that our multi-task model consistently outperforms baselines in terms of f-measures on different datasets. Take CCKS2017 as an example, the single-task model for NES and NER could obtain 91.80% and 91.65% in f-measures on CCKS2017, respectively. The multi-task model achieve 0.19% improvement over single-task model for NER task. This illustrates the effectiveness of our multi-task model. Our method consistently outperforms single-task model, because the addition of a secondary task makes the CRF to obtain more relevant feature information from the network. And this secondary task could improve model performance to get 91.84% in f-measures on CCKS2017. We found that multi-task architecture is generally preferable to single-task architecture, which is consistent with previous research [41].

### Performance of our model for rare entities

In order to evaluate the effect of dictionary information on processing rare entities, we conducted a comparison experiments in terms of recall between our method and two basic models (Baseline1 and Baseline2). The rare entities indicate that they appear in the training set not more than three times, i.e., occurence number $\in \{0,1,2,3\}$.

**Table 10:** Comparative performance (recall) of different methods for rare entities

Method	0	1	2	3
*CCKS2017*				
BERT(Baseline1)	51.47	69.29	81.36	90.34
FT-BERT(Baseline2)	53.62	71.36	82.54	90.97
Our	**53.92**	**76.65**	**85.31**	**91.37**
*FCCd*				
BERT(Baseline1)	50.37	59.47	71.36	84.91
FT-BERT(Baseline2)	52.56	60.38	73.82	84.75
Our	**55.97**	**62.74**	**77.47**	**85.11**

The best result is in bold

Table 10 shows the comparative results in terms of recall. From the table, we can see that our method could achieve higher performance for the unseen entities (non-existent in the training set) compared with the Baseline1 and Baseline2. As for the rare entities (occurrence number $\in \{1, 2, 3\}$), the average recall of our method in FCCd dataset is 70.32%, which is about 3.10% higher than other two methods in average performance. It shows that dictionary information are very important for the recognition of rare and unseen entities. In addition, the impact of dictionary information on model performance decreases as the number of occurrences of entities increases. This is mainly because the more the entity appears in the training set, the better the performance of the model is.

### Performance of our model for different entities types

We further investigate the influence of our method on different categories of clinical entities, we list the experimental results in Table 11. Our method performs well on some categories, such as “Symptom” and “Exam” in CCKS2017, “Independent symptoms”, “Describe symptoms” and “Anatomy” in FCCd. However, its performances were not very well on some categories, such as “Treatment” and “Disease” in CCKS2017. The main reason is that a large number of discontiguous entities are in “Treatment” and “Disease” types. We believe that if a more complete dictionary is provided, better performance could be obtained.

**Table 11:** Performances of our model on each category of entity

Dataset	Entity type	Precision	Recall	F-measure
CCKS2017	Symptom	96.87	97.12	96.99
	Disease	86.32	79.61	82.83
	Exam	94.13	93.81	93.97
	Treatment	82.69	83.73	83.21
	Anatomy	89.67	88.03	88.84
	Average	89.93	88.46	89.16
FCCd	Anatomy	87.03	86.63	86.83
	Operation	86.32	86.03	86.17
	Drug	87.86	85.41	86.62
	IndeSym	88.37	87.58	87.97
	DesSym	87.32	87.02	87.17
	Average	87.38	86.53	86.95

From the above results, we can see that multi-task learning framework could improve the performance on both datasets. The fine-tuned BERT and radical features are very useful in clinical NER tasks. And a complete and delicate dictionary could also help the model to improve performance.

## Discussion

The experimental results showed that our method could effectively identify clinical named entities in Chinese EMRs, and significantly better than other baseline methods. In this section, we analyze the experimental results to illustrate the main reasons that our method can achieve better performance.

We present a novel multi-task deep neural network framework with external dictionary, which can make use of the mutual benefits between entities recognition and segmentation in a more advanced and intelligent way. First, our method benefits from general representations of both tasks provided by multi-task framework. Second, we trained a pre-trained BERT on a large Chinese EMRs which obtain better character representations comparing to traditional methods; In addition, our method can successfully integrate the additional dictionary and radical information into the neural network. Since the dictionary contains rare and unseen entities. Compared with former methods, our method could handle these entities better. Experimental results demonstrated the usefulness of external knowledge and show some promising results from our initial attempt to make use of dictionary information and radical-level features.

An error analysis was done. Firstly, long entity were often not recognized. For instance, “  ” (intestine) is extracted as anatomy entity and “  ” (extranodal mucosa) is considered as symptoms entity, but the correct named entity is “  ” (extranodal mucosa of ileocecal region of intestine). Moreover, there are many irregular entities in clinical texts. For example, the prediction is “  ” (right renal), “  ” (liver), while the correct terms are “  ” (cyst of the upper pole and intermediate of the right kidney), “  ” (multiple metastases to liver and lung). Thirdly, some clinical entities seldom appear in training dataset, so it is difficult to be extracted, such as “  ” (dull pain).

**Fig. 4:**
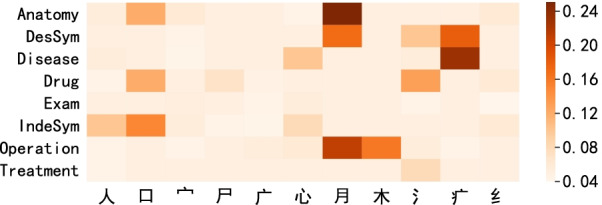
Distribution of representative radicals in three datasets

We experimentally studied the distribution of Chinese radicals in different clinical entities. In order to show the effectiveness of different Chinese radicals, the visualized results are shown in Fig. 4. The radical “  ” often appears more in anatomy, operation, describing symptoms entities. And radical “  ” (the meaning of aching) is more related with diseases , it is often in symptoms entities. The same radicals usually have similar semantic meaning, it is helpful to extract the different entities.

## Conclusion

This paper presents a novel multi-task neural network model for Chinese clinical NER task. It incorporates the dictionary and Chinese radical information to multi-task neural network. Since the dictionary contains rare entities, our proposed approach could process them better than former methods. The evaluation was performed on three datasets. We found that incorporating the dictionary information into the model could improve performance. In future work, we intend to further investigate on how to apply dictionary-based multi-task learning method to recognize nested entities in clinical texts, as well as applications of the proposed model in other related NLP tasks.

## Data Availability

The EHR dataset referenced in this paper comes from hospital, which are not publicly available due to the privacy of patients. The public datasets used and/or analyzed are available from the corresponding author (CM) on reasonable request.

## Abbreviations

EMRs: Electronic medical records
NLP: Natural language processing
NER: Named entity recognition
NES: Named entity segmentation
CRF: Conditional random fields
BiLSTM: Bi-directional Long Short-Term Memory
CNN: Convolution neural network
RNN: Recurrent neural network
BERT: Bidirectional Encoder Representations from Transformers
DicMT: Dictionary-based multi-task neural network model
